# Downregulation of barley ferulate 5-hydroxylase dramatically alters straw lignin structure without impact on mechanical properties

**DOI:** 10.3389/fpls.2022.1125003

**Published:** 2023-01-16

**Authors:** Reza Shafiei, Matthew Hooper, Christopher McClellan, Helena Oakey, Jennifer Stephens, Catherine Lapierre, Yukiko Tsuji, Geert Goeminne, Ruben Vanholme, Wout Boerjan, John Ralph, Claire Halpin

**Affiliations:** ^1^ Division of Plant Sciences, School of Life Sciences, University of Dundee at the James Hutton Institute, Dundee, United Kingdom; ^2^ Faculty of Sciences, School of Agriculture, Food and Wine, University of Adelaide, Adelaide, SA, Australia; ^3^ Cell And Molecular Sciences, James Hutton Institute, Dundee, United Kingdom; ^4^ INRAE AgroParisTech, Université Paris Saclay, IJPB, Versailles, France; ^5^ Department of Biochemistry, University of Wisconsin-Madison, Madison, WI, United States; ^6^ Department of Energy’s Great Lakes Bioenergy Research Center, The Wisconsin Energy Institute, University of Wisconsin-Madison, Madison, WI, United States; ^7^ VIB-UGent, Metabolomics Core, Ghent, Belgium; ^8^ Department of Plant Biotechnology and Bioinformatics, Ghent University, Ghent, Belgium; ^9^ VIB-UGent, Center for Plant Systems Biology, Ghent, Belgium

**Keywords:** ferulate 5-hydroxylase, lignin, barley, RNAi, S/G, straw, biosynthetic pathway, bioeconomy

## Abstract

Barley is a major cereal crop for temperate climates, and a diploid genetic model for polyploid wheat. Cereal straw biomass is an attractive source of feedstock for green technologies but lignin, a key determinant of feedstock recalcitrance, complicates bio-conversion processes. However, manipulating lignin content to improve the conversion process could negatively affect agronomic traits. An alternative approach is to manipulate lignin composition which influences the physical and chemical properties of straw. This study validates the function of a barley ferulate 5-hydroxylase gene and demonstrates that its downregulation using the RNA-interference approach substantially impacts lignin composition. We identified five barley genes having putative ferulate 5-hydroxylase activity. Downregulation of *HvF5H1* substantially reduced the lignin syringyl/guaiacyl (S/G) ratio in straw while the lignin content, straw mechanical properties, plant growth habit, and grain characteristics all remained unaffected. Metabolic profiling revealed significant changes in the abundance of 173 features in the *HvF5H1*-RNAi lines. The drastic changes in the lignin polymer of transgenic lines highlight the plasticity of barley lignification processes and the associated potential for manipulating and improving lignocellulosic biomass as a feedstock for green technologies. On the other hand, our results highlight some differences between the lignin biosynthetic pathway in barley, a temperate climate grass, and the warm climate grass, rice, and underscore potential diversity in the lignin biosynthetic pathways in grasses.

## 1 Introduction

Lignocellulosic feedstocks are attractive commodities for producing a variety of bio-products due to their renewable nature and smaller environmental footprint compared to petrochemicals. However, conversion of lignocellulosic biomass into high value bio-products and second-generation biofuels is challenging, partly due to the presence, heterogeneity, and structural complexity of lignin. This has led to a focus on researching the biosynthesis, properties, and manipulation of lignin to increase both fundamental understanding, and to explore opportunities for biomass improvement.

Lignin is produced *via* the phenylpropanoid pathway which starts with the enzyme phenylalanine ammonia-lyase (PAL) and ultimately synthesizes the H (*p*-hydroxyphenyl), G (guaiacyl), and S (syringyl) monolignol units. Lignification begins when these monolignols are oxidised by cell-wall laccases and/or peroxidases resulting in radicals that are combinatorially coupled through ether or carbon-carbon bonds leading to assembly of lignin polymers. The outcome of the polymerization is heavily influenced by the type and amount of phenolic monomers that are translocated into the cell wall and that are competent for free-radical polymerization (for review, see Tobimatsu et al., 2013). Grass lignin is mainly made up of S and G units, with H units constituting a small but significant percentage. Lignin composition influences the physical and chemical properties of biomass feedstocks and has been one of the key targets of cell wall engineering studies (Halpin, 2019).

Recent research has highlighted some of the unique features of the lignin pathway in grasses compared to dicots. In dicots, lignin biosynthesis starts with the deamination of phenylalanine (Phe) into cinnamic acid and subsequent conversion to *p*-coumaric acid (*p*CA) *via* cinnamate 4-hydroxylase (C4H) activity. Grasses are able to synthesize *p*CA from Phe or tyrosine (Tyr) using bifunctional phenylalanine/tyrosine ammonia-lyases (PTAL), with the TAL activity contributing to nearly half of the cell wall total lignin in Brachypodium (Barros et al., 2016). The metabolites derived from Tyr seem to be preferentially incorporated into S lignin units and into cell-wall-bound *p*CA, another characteristic feature of grass cell walls (Withers et al., 2012; Barros et al., 2016). Some of that wall-bound *p*CA is attached to lignin through the involvement of *p*-coumaroyl-CoA:monolignol acyltransferase (PMT) in the acylation of a proportion of monolignols to produce *p*-coumaroylated S monolignol conjugates (Marita et al., 2014; Petrik et al., 2014). The conventional lignin biosynthetic pathway enzyme, ferulate 5-hydroxylase (F5H, also known as coniferaldehyde 5-hydroxylase or Cald5H), is essential to S lignin biosynthesis in dicots, but relatively few studies have examined its role in grasses. Recent work in rice has suggested that F5H may only catalyse production of the nonacylated portion of lignins in grasses (Takeda et al., 2019a). This has led to the hypothesis that a grass specific lignin pathway may exist for the production of *p*-coumaroylated S lignin which is independent of the co-existing conventional lignin biosynthesis pathway (Takeda et al., 2019a; Barros and Dixon, 2020). However, the genes and enzymes of the hypothetical grass specific lignin pathway remain to be identified and it is still unclear whether this pathway, if it exists, is a feature of lignin biosynthesis unique to rice or common to other grasses. Consequently, the role of F5H in dictating the overall S lignin content of grasses is still an open question.

The proportions of G and S units in lignin critically influence the structure of the polymer and the ease of enzymatic biomass processing to release sugars (saccharification) for downstream uses. In the conventional S lignin biosynthetic pathway, the C5 position on the G-lignin precursor units (coniferaldehyde or coniferyl alcohol) is hydroxylated and methylated by F5H and caffeic acid *O*-methyltransferase (COMT) enzymes, respectively. *F5H* knockout plants of Arabidopsis lack S-lignins (Chapple et al., 1992). The availability of C5 on G-lignin precursors can lead to pretreatment-recalcitrant β–5 or 5–5’ linkages within lignin and trigger inter-chain connections during lignin polymerization, such that G-rich lignins are more chemically complicated with larger molecular weights and higher melting points compared to S rich lignins (Vanholme et al., 2010; Ralph et al., 2019). Structurally, a lignin polymer that is extreme in its content of S units, methoxylated at both C3 and C5, is mainly composed of more homogeneous linear chains, which are shorter relative to H- or G-rich lignins and have a high proportion of chemically-labile β–*O*–4 bonds (Stewart et al., 2009; Vanholme et al., 2010). Many studies in various dicot species in which F5H expression has been manipulated illustrate that reduced *F5H* expression reduces S lignin units and can lower sugar release after biomass pretreatment, whereas increased *F5H* expression increases S lignin units and may improve sugar release (e.g., Ciesielski et al., 2014; Fan et al., 2020). However, the few studies in grasses that link F5H expression and S lignin content to saccharification efficiency have yielded contradictory results. Research performed on Brachypodium yielded results consistent with those from dicots (Sibout et al., 2017), whereas work on switchgrass revealed no change to saccharification on F5H up- or down-regulation (Wu et al., 2019), and work on sugarcane surprisingly indicated that reduced F5H expression and reduced S units in lignin might increase sugar release upon saccharification after mild acid pretreatment (Bewg et al., 2016). The roles of F5H and S lignin content in influencing saccharification and biomass processing in grasses after various pretreatments therefore remains an open question and it is possible that not all grasses behave in the same way.

In order to clarify both the role of F5H in influencing the S lignin content of grass lignins, and to explore the impact of *F5H* downregulation on saccharification efficiency, research is needed in a wider variety of grasses. Barley (*Hordeum vulgare*) is the fourth largest cereal crop globally and a close relative of wheat (*Triticum aestivum*), the second most important global crop. Together, barley and wheat crops are the greatest source of straw biomass in temperate regions. In this study, we identified five *F5H* genes in barley and by RNAi downregulation we validated that one of these genes, *HvF5H1*, plays a dominant role in S lignin production and in determining the lignin S/G ratio in straw. Subsequently, we studied the impact of *F5H* downregulation on saccharification recalcitrance, agronomic traits, and straw mechanical properties. Our data suggest differences in overall S lignin pathways between barley and what has been reported for rice and highlights potential diversity in lignin biosynthesis in grasses with implications for straw improvement strategies.

## 2 Results

### 2.1 Barley *F5H* gene discovery

F5H is a cytochrome P450 monooxygenase enzyme and a member of the CYP84 family (Meyer et al., 1998). Putative barley F5Hs were shortlisted in two steps. First, we blast-searched the barley whole-genome Morex assembly and used multiple sequence alignment to identify proteins that had all the conserved structures and signature motifs in F5Hs as described previously (Meyer et al., 1996; Bak et al., 2011). Next, using phylogenetic analysis, candidate sequences from barley were compared with functionally characterised *F5H* genes in Arabidopsis (Meyer et al., 1998) and grasses including rice (Takeda et al., 2017), switchgrass (Wu et al., 2019), and sugarcane (Bewg et al., 2016). This led to identification of five putative *F5H* genes in barley, named *HvF5H1-5* (HORVU1Hr1G047220, HORVU3Hr1G014930, HORVU2Hr1G109440, HORVU7Hr1G084500, and HORVU2Hr1G092360, respectively). The derived amino acid sequences of these genes are shown in **Figure S1**.

**Figure 1:**
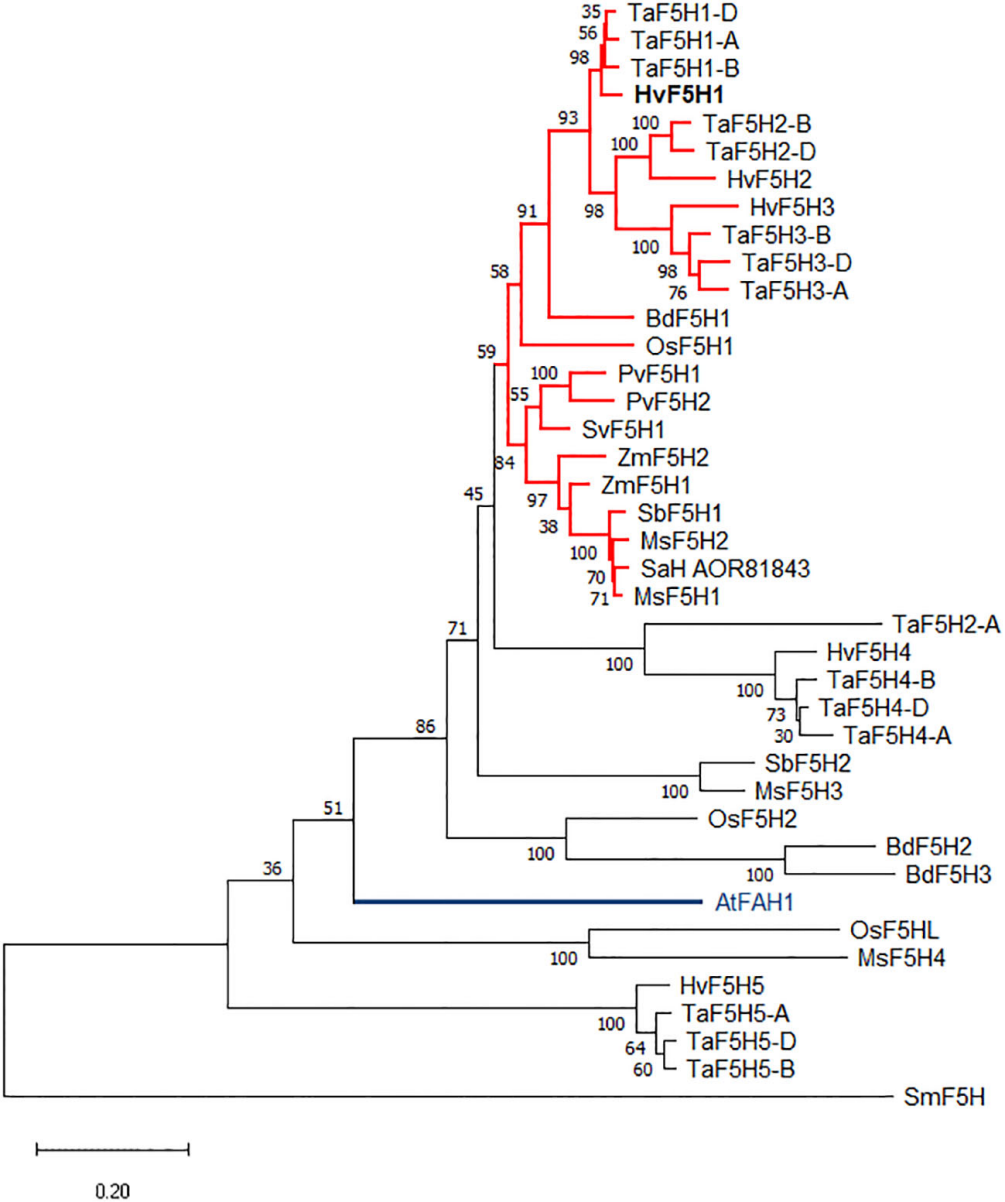
The rooted phylogenetic tree was constructed using the Maximum Likelihood method and JTT matrix-based model, with the F5H protein sequences from *Selaginella moellenodorffii* (Sm), barley (Hv), wheat (Ta), rice (Os), maize (Zm), Brachypodium (Bd), *Miscanthus* (Ms), sugarcane (SaH), sorghum (Sb), switchgrass (Pv), green foxtail (Sv), and Arabidopsis (At). The Arabidopsis *AtFAH1* is highlighted in blue. All functionally characterised grass F5H proteins with activity in lignin biosynthesis belong to the red coloured clade although not all the proteins in this clade have been characterised. Bootstrapping values from 100 trials are shown next to the branches. The tree is drawn to scale and the scale bar represents 0.2 amino acid substitutions per site. See **Table S1** for the accession numbers and further information.

A phylogenetic tree was constructed using the Maximum Likelihood method and a JTT matrix-based model that shows the relationships between these five putative barley F5H amino acid sequences and those from other C3 and C4 grasses (**Figure 1**). Spike moss (*Selaginella moellenodorffii*) smF5H was brought into this analysis as an outlier (Weng et al., 2008). The phylogenetic tree shows that the majority of putative F5H proteins in grasses group closely together, forming a large cluster that encompasses all previously functionally characterised grass F5Hs (**Figure 1** red clade). This cluster includes protein sequences for barley *HvF5H1*, *HvF5H2*, and *HvF5H3* genes, hypothetically supporting a direct role for these in lignin biosynthesis. The presence of three barley F5Hs in this clade represents an expansion of the gene family in barley compared to some other grasses that have only one F5H in the clade (e.g., Brachypodium and rice). By comparison, wheat has two or three TaF5H counterparts for each barley sequence representing the three ancestral genomes that compose modern hexaploid wheat. Collectively these data suggest duplications of the *F5H* gene in barley and wheat and possibly all Triticeae after their divergence from the Brachypodieae lineage. Predictably, *Sm*F5H and *At*FAH1 proteins appeared to be less closely related to the grass F5Hs and are located in isolated distant clades. We obtained similar phylogenetic results using the UPGMA (Unweighted Pair Group Method with Arithmetic mean) and Neighbour Joining methods.

The phylogenetic tree also encompasses five other small clades of amino acid sequences that carry F5H signature motifs but have unknown functions potentially distinct from lignin biosynthesis. The *OsF5H2* (CYP84A6) gene was reported to be expressed in rice ovary 3 days after flowering (Takeda et al., 2017) and in mature leaves (Kim et al., 2006) but is not considered to be an essential gene in lignifying tissues (Takeda et al., 2017). Similarly, in Arabidopsis, duplication and neofunctionalisation of *AtFAH1* has produced a paralogous gene, *AtCYP84A4*, of which the corresponding enzyme has a widely altered catalytic activity in α-pyrone metabolite biosynthesis (Weng et al., 2012).

The *Hv*F5Hs show 60-65% amino acid sequence identity to *At*FAH1 and 65-81% identity to orthologs in the Poaceae family. Out of the five identified F5H homologs in barley, *Hv*F5H1 shared the highest amino acid sequence identity to the functionally characterised F5Hs from Arabidopsis (*At*FAH1, 65%), rice (*Os*F5H1, 80.7%), switchgrass (*Pv*F5H1, 80.8%), and sugarcane (*SaH*_AOR81843, 81%). Furthermore, barley RNAseq data (Mascher et al., 2017) revealed that the expression pattern of *HvF5H1* is characteristically different from the other *HvF5H*s (**Figure S2**). *HvF5H1* is the only *HvF5H* expressed to substantial levels with the highest transcript abundances found in lignifying tissues as well as leaves (**Figure S2**). This is particularly important as it demonstrates *HvF5H1* is highly active in lignocellulosic biomass but not in the grain or inflorescence tissues. *HvF5H3* shows some expression only in embryo and roots, whereas *HvF5H4* expression was found only in roots. Very limited expression was detected for *HvF5H2* or *HvF5H5* in any of the 16 tissues presented in the dataset. The expression data combined with phylogenetic analysis clearly suggest the *HvF5H1* gene as the strongest candidate for an involvement in lignin biosynthesis.

### 2.2 Downregulating *HvF5H1* in barley

To manipulate lignin composition and structure and investigate the influence of *Hv*F5H1 on S unit biosynthesis, we used the RNAi approach to produce transgenic plants with suppressed *HvF5H1* expression. A fragment of 669 bp (**Figure S3**) was used to produce a hairpin inverted repeat sequence in the plasmid pBract207 under the control of the constitutive maize ubiquitin promoter. Subsequently, 23 T0 independent transgenic plants were produced following the transformation of the Golden Promise cultivar (**Figure S4A**). Southern blot analysis identified plants with a single T-DNA locus and qPCR was employed to assay plants for reduced *HvF5H1* expression relative to wild-type plants (**Figure S4B**). The qPCR was carried out on samples of second internode taken at growth stage 32 on the Zadoks scale (Zadoks et al., 1974). Of 10 T0 plants that had a single insertion site of the T-DNA locus, nine also exhibited at least 60% reduction in *HvF5H1* expression compared to the wild type and greater than 67% reduction compared to the empty vector (EV) plants; the best four lines showed approximately 95% or more reduction in *HvF5H1* expression. From the pool of nine T0 plants, we selected seven lines to develop into the T1 generation. These lines were named B, I, K, M, T, W, Y. T1 generation plants were screened to identify *HvF5H1*-RNAi homozygote and azygote plants that had lost the transgene through segregation. Consequently, we selected three independent lines B, T, and W, to analyse further in the T2 generation. For this purpose, homozygous RNAi T2 plants along with control lines and wild type were grown in the greenhouse in a Randomised Complete Block design with five replications. Levels of *HvF5H1* transcript reduction were evaluated to be 90% for line B, 88% for line W and 93% for line T (**Figure S5**). Using Mäule staining, the second internodes of the T2 plants were investigated for a change in S lignin relative to control plants. Whereas all control plants (wild type, empty-vector controls, and azygote plants that had lost the RNAi transgene through segregation) showed bright red staining in lignified tissues, in the RNAi lines the vascular bundles and cortical fibre tissues exhibited more of a brown colour, indicating a reduction in the proportion of S lignin (**Figure S6**).

### 2.3 Altered lignin composition in downregulated plants

**Figure 2:**
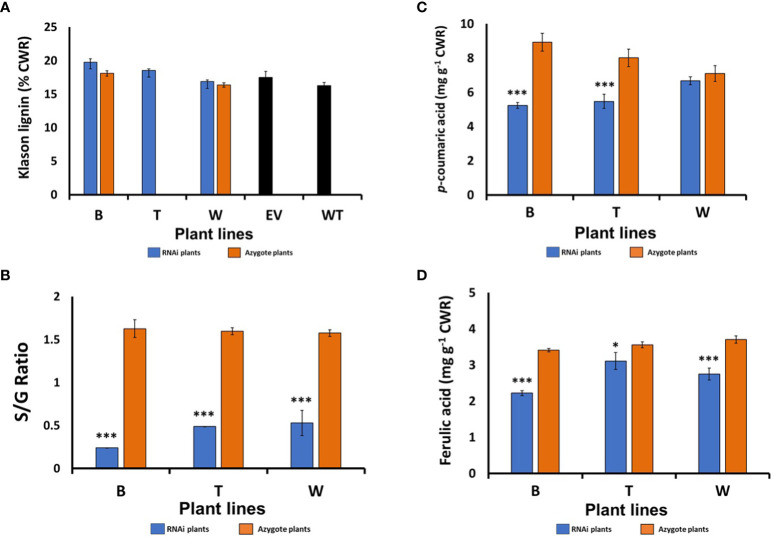
Cell wall compositional analysis of the senesced barley straw from the T2 generation *HvF5H1-*RNAi lines and controls: **(A)** Klason lignin content, **(B)** S/G ratio, **(C)** Ester-linked *p*‐coumaric acid (*p*CA), **(D)** ester-linked ferulic acid (FA), as measured by mild alkaline hydrolysis. Lines marked with * & *** are significantly different from their controls which were the respective azygote plants in the case of lines B and W; for line T where no azygous controls were available, comparisons were made with empty vector (EV) plants for Klason lignin or with all azygote plants for S/G ratio and ester-linked *p*CA and FA, Student’s t‐test *P*<0.05 and *P*<0.001, respectively for * and ***. The error bars represent standard errors between 3-7 biological replicates (i.e. independent plants). B, T, and W; RNAi lines; EV: Empty vector; WT: wild type.

To investigate the consequence of suppressed *HvF5H1* expression on lignin content and composition/structure, extract-free cell wall residue (CWR) was prepared from the main tiller of fully-senesced T2 generation RNAi homozygote plants and related control plants. We removed leaf sheaths from the tillers as their cell wall structure and lignin content differs from culms. Klason lignin determinations on tiller CWR showed no significant differences in Klason lignin content between the *HvF5H1*-RNAi plants and the controls (**Figure 2A**).

Analytical thioacidolysis and 2D NMR (HSQC) were employed to study lignin composition of *HvF5H1* RNAi lines in more detail. Thioacidolysis degradation releases H, G and S thioethylated monomers only from H, G and S lignin units linked by β-aryl ether (β–O–4) bonds to reflect relative abundance of these monomers (Lapierre et al., 1986). The thioacidolysis yield (in µmol/g Klason lignin) was reduced by up to 27% in the *HvF5H1*-RNAi lines indicating a reduction in the proportion of units linked by β-aryl ether bonds. The S/G ratio of thioacidolysis released monomers in *HvF5H1*-RNAi lines decreased by up to 85% from 1.62 in line B azygotes to 0.24 in the corresponding RNAi plants (*P*<0.01; **Figure 2B**) reflecting a shift from 60% S units and 37% G units in lignin in azygote plants to 19% S units and 78% G units in lignin in RNAi plants (**Figure S7**). Changes in RNAi lines W and T were similar but slightly less severe. These findings are in line with F5H’s role in hydroxylating G-lignin precursor units (coniferaldehyde or coniferyl alcohol) to produce S lignin monomers. Our analyses also revealed a significant reduction (*P* < 0.01) of 28% and 41% in the amount of ester-linked *p*CA released by mild alkaline hydrolysis in *HvF5H1*-RNAi lines T and B respectively (**Figure 2C**), although the 6% reduction in line W was not significant. Ester-linked FA was also significantly reduced in all RNAi lines (**Figure 2D**). Reductions in ester-linked *p*CA are consistent with the reduction of S lignin given that much of the cell wall *p*CA in grasses is ester-linked to S units (Ralph et al., 1994).

**Figure 3:**
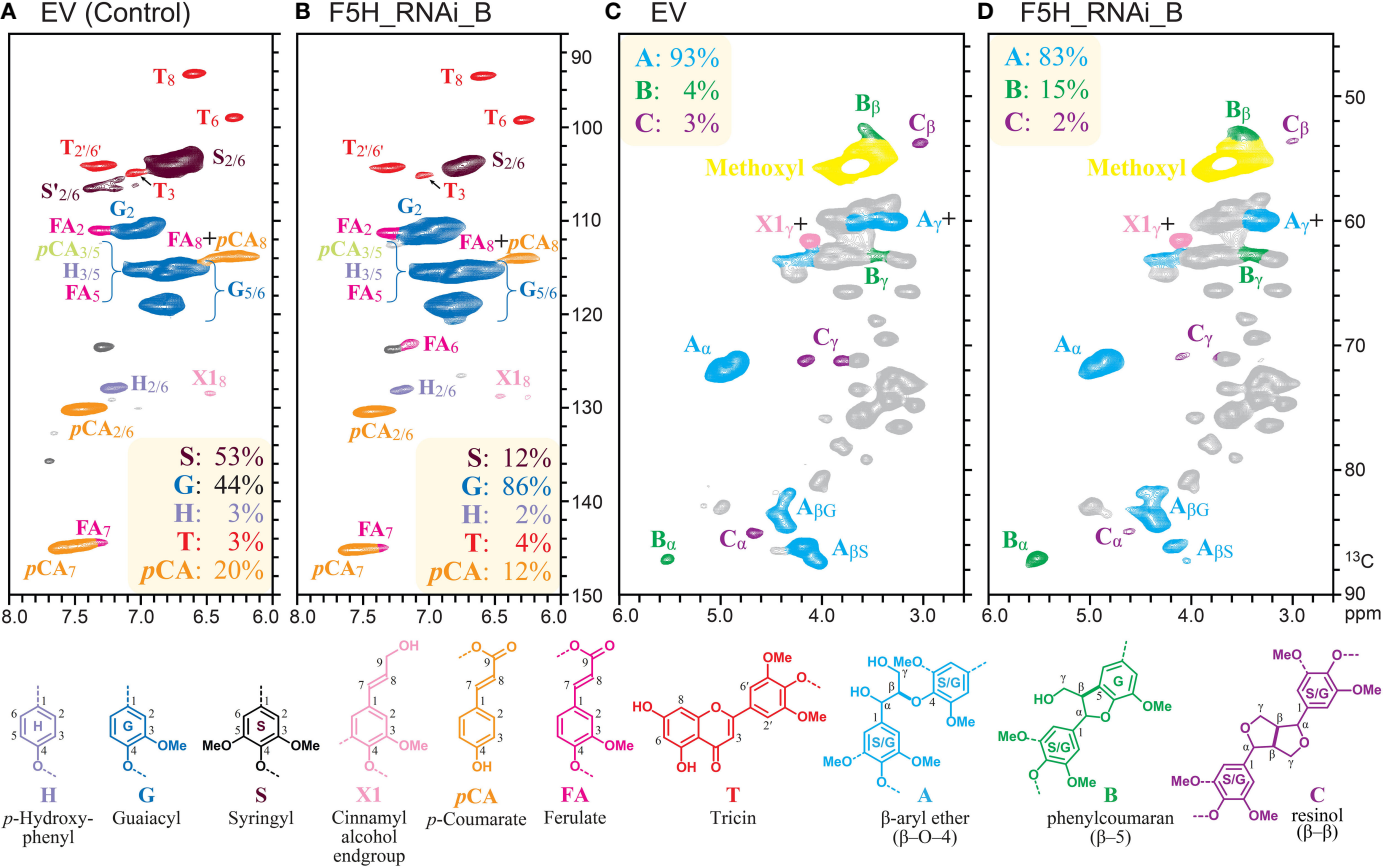
Aromatic and aliphatic subregions of 2D-NMR (HSQC) spectra of stem cell walls from *HvF5H1*-RNAi plants (T2 generation) and EV. Summary data in the boxes color-coded to match their respective assignments, and represent percentages of; **(A, B)** major lignin subunits to the total amount of H, G, and S units; and **(C, D)** characterised inter-monomeric bonds to the total number of the lignin interunit linkages.

A 2D-NMR (HSQC) analysis of line B cell wall material further verified the lignin compositional changes in the *HvF5H1*-RNAi plants that had been demonstrated through analytical thioacidolysis (**Figures 3A, B**). The greatest alterations were observed in S and G units. The G units in the RNAi line increased proportionally to 86% compared to 44% in the control. On the other hand, suppression of *HvF5H1* led to reduction of S unit content to 12% in the RNAi plant compared to 53% in the control plant. The H-unit content slightly decreased in the RNAi plant (2%) relative to control (3%). In agreement with thioacidolysis, NMR detected 12% of *p*CA in the RNAi plant versus 20% in the control and 4% tricin in the RNAi plant versus 3% in the control; such mobile end-units are, however, over-quantified by NMR (Mansfield et al., 2012).

The aliphatic subregion of the 2D-NMR spectra shows the signals that are derived from interunit linkages in the CWR lignin polymer (**Figures 3C, D**). In both RNAi and control plants, the dominant units are β-aryl ethers followed by substantially lower percentages of phenylcoumaran (β–5) and resinol (β–β). The proportion of β-aryl ether bonds, which are the main intermolecular linkages in native lignin, were reduced from 93% in control to 83% in the RNAi line, consistent with the thioacidolysis yield reduction which also indicated reduced β-aryl ether linkages. On the other hand, the share of phenylcoumaran linkages in the RNAi line increased to 15% compared to 4% in the control. There was little change in the proportion of resinol bonds. These data are all consistent with the higher proportion of G units in the *HvF5H1*-downregulated plants.

### 2.4 Metabolic profiling of *HvF5H1* RNAi lines

To study the impact of *HvF5H1* suppression on intracellular phenolic metabolites, we sampled the first three internodes of plants from two *HvF5H1*-RNAi lines (lines B and W) to compare with wild-type and EV control plants. UHPLC-MS was employed to analyse phenolic metabolites extracted from the internodes. Initially, our analysis detected 7486 features with an abundance of more than 100 counts in at least one sample. Features were selected for further investigation if their abundance was significantly (*P*<0.01) different from the controls for both *HvF5H1*-RNAi transgenic lines. This led to the identification of 173 features (i.e., *m/z* traces*)* with at least three-fold change in both RNAi lines compared to control values. Of these, 95 features showed higher intensity in the *Hv*F5H1 RNAi plants whereas 78 *m/z* traces exhibited lower intensity (**Table S2**). Taking into account the accurate *m/z*, retention time, and MS/MS fragmentation, we characterised 47 metabolites. The 21 identified metabolites with higher intensity in the RNAi lines were mainly small oligomers composed of G units only, or in combination with units derived from ferulic acid or tricin. In contrast, the metabolites with reduced abundance were sinapyl alcohol, three conjugates with syringic acid, and 22 di- and tri-lignols containing at least one S unit.

### 2.5 Transcriptomic profiling in *HvF5H1*-RNAi lines

We performed a transcript profiling in order to evaluate the effect of *HvF5H1* downregulation on the expression of other genes in stems. For this purpose, the first three internodes were sampled from two *HvF5H1*-RNAi lines (lines B and W) and compared with wild-type and EV control. To identify differentially regulated genes, expression data was first log-normalised and the genes that were significantly (*P*<0.01) different between the *HvF5H1*-RNAi lines and both controls were identified. Then we shortlisted genes that were up- or down-regulated greater than three-fold. In the process, probes that had a significant (*P*<0.001) expression difference between the wild-type and empty vector controls were excluded due to lack of consistency. Subsequently, fourteen genes were identified as upregulated and four genes as downregulated (**Table S3**) in the RNAi lines compared to controls. *HvF5H1*, the targeted gene, was downregulated 4.7-fold. Of the other *HvF5H* genes, *HvF5H2* and *HvF5H5* expression was not detected by transcript profiling and expression of both *HvF5H3* and *HvF5H4* remained unchanged. The top-downregulated gene (HORVU2Hr1G052170) belonged to the alpha/beta hydrolase superfamily and was downregulated about 7-fold.

The most differentially overexpressed genes in the RNAi lines were a DNA/RNA helicase which is involved in meiotic crossover formation (HORVU4Hr1G049830, 22-fold), and a hydroxyjasmonate sulfotransferase (HORVU2Hr1G117700, 15-fold). An NBS LRR disease resistance gene along with a MYB transcription factor were upregulated 12.4 and 6.6-fold, respectively, in the *HvF5H1*-RNAi lines.

### 2.6 *HvF5H1* downregulation did not affect growth habit or straw characteristics

**Figure 4:**
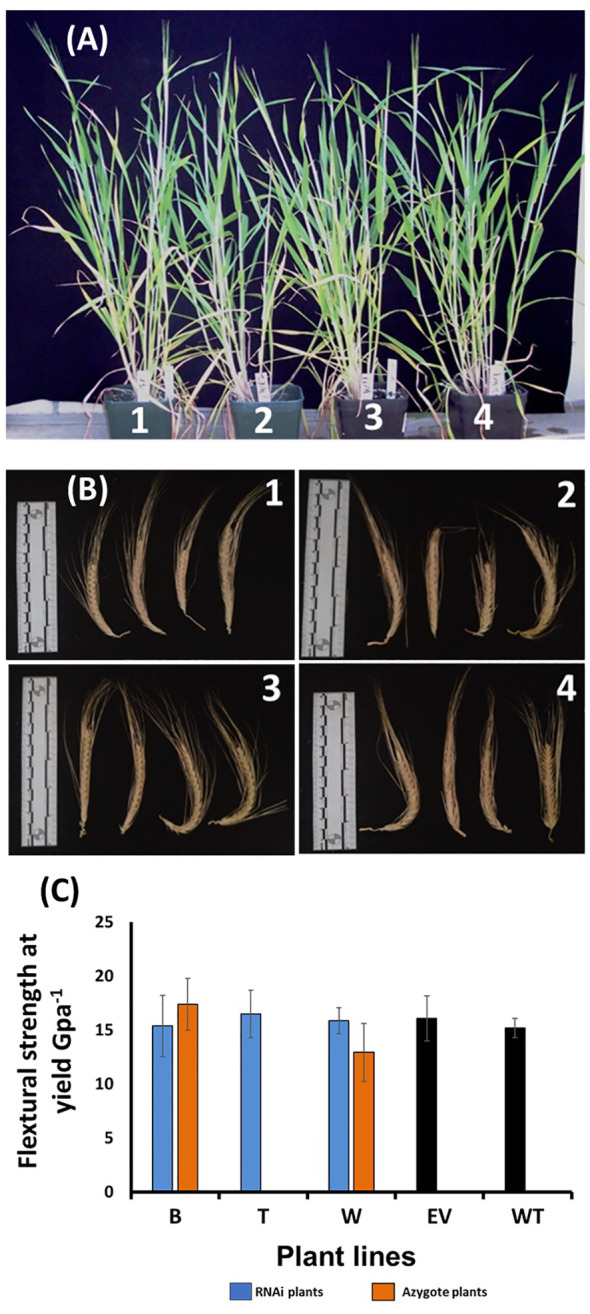
Phenotypic characterization of the *HvF5H1*-RNAi lines and controls from the T2 generation; **(A)** plant stature and **(B)** spikes at harvest time, where 1,2,3,4 are wild type, empty vector, azygote and *HvF5H1* RNAi plants respectively; **(C)** mechanical strength of the second and fourth internodes sampled from main tillers of the ripe senesced straw; No significant differences were identified (P < 0.05) following Student’s t‐test. B, T, W are RNAi lines and B and W have corresponding azygote lines. For line T where no azygotes were available, empty vector and wild type plants were used as controls. The error bars represent standard errors between biological triplicates (i.e. independent plants).

Given the considerable modification in lignin structure of the *HvF5H1*-RNAi plants, we phenotyped them for a variety of agronomic traits to determine whether such dramatic changes to lignin might impact directly on crop productivity. Visually, the *HvF5H1*-RNAi lines did not exhibit noticeable phenotypic differences compared to the control plants (**Figure 4A**). Plants were phenotyped for shoot height, tiller number, straw biomass, spike number, and seed characteristics including seed length, width, and area and thousand-grain weight (TGW) (**Table S4**). Employing ANOVA, none of these traits in the RNAi lines was significantly different from the ones in control plants (*P*<0.05).

Lignin modification could impact straw strength and potentially the plant’s resilience or lodging resistance, with indirect effects on productivity and yield. We therefore tested the mechanical properties of stems sampled from the RNAi lines at harvest time by quantifying the maximum stretching force straw can tolerate before it breaks, i.e. the ‘Flexural Strength Stress at Yield’. For this purpose, we sampled second and fourth internodes from the main tiller and employed an Instron Universal Testing System for precision phenotyping. Predictably, in each genotype significantly (*P*<0.05) less force was required to break the fourth internode compared to the corresponding second internode. However, we did not find a significant difference between the RNAi lines and the controls (**Figure 4C**, **Table S5**) when either the second or fourth internodes were analysed, suggesting that the S/G ratio reduction did not impact the mechanical strength of barley straw.

### 2.7 Saccharification of *Hv*F5H1-RNAi lines

**Figure 5:**
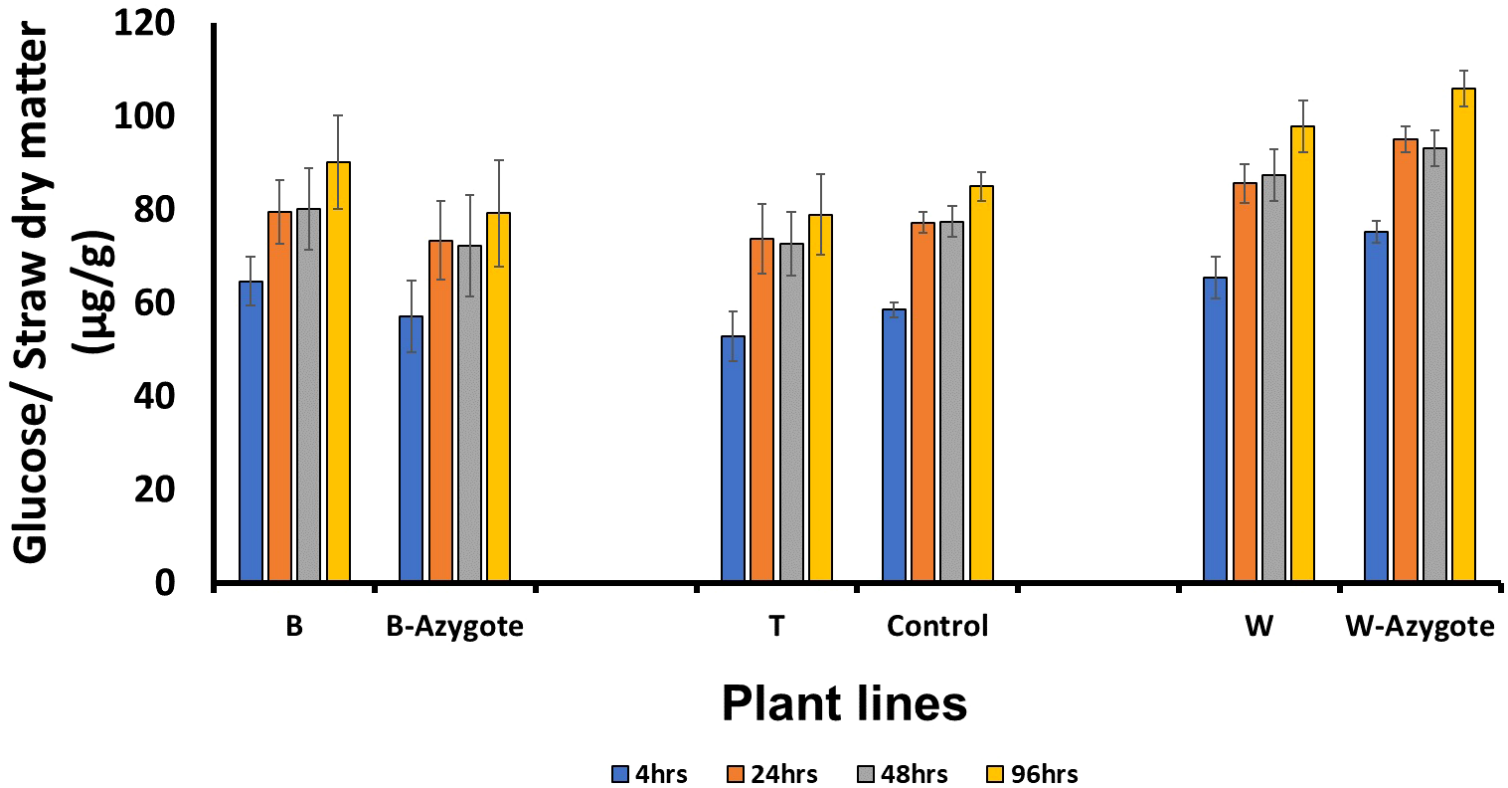
Saccharification following hot water pretreatment. No significant differences (P < 0.05) were identified following Student’s t‐test. B, T, W are RNAi lines; B-azygote, W-azygote are corresponding azygote lines; for line T where no azygotes were available, empty vector and wild type plants were used as controls. The error bars represent standard errors between biological triplicates (i.e. independent plants).

To investigate the consequence of lignin structural changes on straw recalcitrance, we carried out saccharification assays after subjecting the straws to hot water pretreatment. The enzymatic digestion phase lasted 96 hours and samples were collected at 4, 24, 48 and 96 hours. However, there was not a significant improvement or deterioration in saccharification efficiency when each RNAi line was compared with its most relevant controls following Student’s t‐test (P < 0.05) (**Figure 5**).

## 3 Discussion

This study demonstrates that expression of *HvF5H1* plays a major role in determining the proportions of S and G units in lignin in barley culm tissues. Transgenic plants with downregulated *HvF5H1* expression have substantially reduced frequency of S units in culm lignin and a correlatively high enrichment in G units compared to control plants. In the RNAi line with the largest shift in lignin composition, S units represented only 19% of the thioacidolysis released units as compared to 60% in the azygote control plants, representing a relative decrease of 68% in S units. Correspondingly, G units increased from 37% of the units released from azygote plants to 78% of the released units in RNAi plants, a relative increase of 111%. Concomitant with the %S decrease and %G increase, the thioacidolysis yield was reduced, which is consistent with the higher proclivity of G units to be involved in resistant interunit bonds such as phenylcoumaran or biphenyl linkages that thioacidolysis does not release monomers from. Our 2D NMR analysis independently confirmed the thioacidolysis lignin data revealing a 73% reduction in S units and a 95% increase in G units overall. Unlike thioacidolysis which releases monomers from lignin units only involved in β-aryl ether units (linked by their characteristic β–O–4-ether bonds), 2D-NMR on unfractionated cell walls can provide information on additional lignin linkages and showed that, although the proportion of β-ether units decreased from 93% in the wild type to 83% in the RNAi line, phenylcoumaran linkages proportionally increased significantly, from 4% in the wild type to 15% in the RNAi line. There was little change in resinol linkages in the RNAi line. These changes to lignin on *HvF5H1* suppression are very consistent with what has previously been seen in dicot species (e.g., Meyer et al., 1998; Reddy et al., 2005) and converse changes (i.e., an increase in the proportion of S units) have been seen when *F5H* is over-expressed (e.g., Meyer et al., 1998; Franke et al., 2000; Stewart et al., 2009). Our analyses also showed significant reductions in ester-linked *p*CA in two *HvF5H1* RNAi lines. Mild alkaline hydrolysis indicated a 28 and 41% reduction in the amount of ester-linked *p*CA in *HvF5H*1-RNAi lines T and B respectively. As much of the cell wall *p*CA in grasses is ester-linked to S units in the lignin, a reduction in esterified *p*CA when S units are reduced is not unexpected, although the proportion of *p*CA that is ester-linked to arabinoxylans would be retained. Again, 2D-NMR confirmed a *p*CA reduction of approximately 40% suggesting that the impact of *HvF5H1* suppression is not restricted to the nonacylated portion of lignin as in rice (Takeda et al., 2019a), but indicates that the *p*-coumaroylated S lignin has been reduced.

In contrast to the many studies on F5H manipulation in dicots, only a few studies address the impact of F5H suppression in grasses. In sugarcane, *F5H* was suppressed by RNAi to varying degrees although 16-94% of control expression remained in four transgenic lines analysed (Bewg et al., 2016). Nevertheless, the best transgenic line had an altered S:G ratio of 48:52 compared to 61:39 in controls, i.e., a 21% decrease in the proportion of S units and a 33% increase in the proportion of G units, changes completely consistent with but less extreme than what we observed in barley. The NMR analysis also detected phenylcoumaran (β–5-linked) units in this transgenic plant but not in the controls (Bewg et al., 2016). Similarly, in *F5H*-RNAi suppressed switchgrass (Wu et al., 2019), plants with approximately 55% reduction in *F5H* expression, by our calculations had an altered S:G ratio of about 32:68 compared to 46:54 in controls, i.e., a 30% decrease in the proportion of S units and a 26% increase in the proportion of G units. In an initial study in rice, *F5H1* suppression by RNAi eliminated over 90% *F5H1* expression but S:G ratio determined *via* thioacidolysis was only reduced from 36:64 in control to 26:74 in the RNAi lines, i.e., a relative decrease of S units by 28% according to Takeda et al. (2017). To determine whether the high proportion of remaining S units was due to residual F5H1 activity, or to the activity of alternative unidentified hydroxylases, the same research group knocked out rice *F5H1* using CRISPR/Cas9 (Takeda et al., 2019a). Surprisingly, plants with presumed total knock-out of *F5H1* still produced considerable amounts of S units in the culm, to approximately 62–70% of the level of wild-type plants. This is very unexpected as an F5H knock-out mutant in Arabidopsis, the *fah1-2* mutant, produces no S lignin (Chapple et al., 1992). Moreover, DFRC lignin analysis of the rice *F5H1* CRISPR knock-out indicated that the changes in G and S units were restricted to the non-*p*-coumaroylated lignin units and units with *p*CA attached were almost unchanged (Takeda et al., 2019a). This discovery prompted the authors and others to propose that rice and possibly grasses in general must have two parallel pathways for making S lignin, only one of which requires F5H1 whereas monolignol units to which *p*CA is attached are produced by an undiscovered alternative pathway (Takeda et al., 2019a; Barros and Dixon, 2020).

Our results throw some of the details of this hypothesis into question at least for barley. Only *HvF5H1* was downregulated in our experiments and transcriptomic data from RNAi plants confirmed that expression of other *HvF5Hs* did not change. Nevertheless, we achieved a substantial decrease of 68-73% in S units using RNAi compared to in rice where there were only 32% and 38% S unit reductions using RNAi and CRISPR knock-outs respectively. The approximately 30% of S units remaining in our barley plants could be explained by the low level of *HvF5H1* expression that remains. The levels of esterified *p*CA in cell walls was also clearly reduced in barley by approximately 30–40% consistent with the reduction in S units and the fact that most *p*CA esterified to grass lignin is found on S units (although a significant proportion is found esterified to arabinoxylan). Consequently, although we cannot conclude that an alternative S lignin pathway does not exist in barley, our results can be explained based on the conventional lignin pathway alone. Taking into consideration the scale of intermolecular bond alterations, the high impact of *HvF5H1* RNAi downregulation on S and G lignin, and the significant reduction of *p*CA, *HvF5H1* is a major gene for S lignin biosynthesis in barley and the conventional lignin pathway clearly dominates.

The changes in lignin in our *HvF5H1*-RNAi plants appear to have no impact on saccharification. This is consistent with most other work in the literature that either shows no change, or a reduction in saccharification, in *F5H*-suppressed plants, and an increase in saccharification when F5H is overexpressed and S lignin proportion increased. Several papers demonstrate that the predominantly G-lignin in the Arabidopsis *fah1* F5H mutant impedes saccharification compared to wild-type lignin after liquid hot water pretreatment (Li et al., 2010) or maleic acid treatment (Ciesielski et al., 2014), although weak alkaline pretreatment reveals no change to saccharification (Smith et al., 2022). On the other hand, *Atf5h1* T-DNA insertion mutants allelic to *fah1* did show an improved saccharification when no pretreatment was used (van Acker et al., 2013). In *Populus tomentosa*, downregulating *F5H* expression by manipulating miR6443, a microRNA that regulates S lignin biosynthesis, increases G units and reduces S units in lignin and lowers sugar yield after saccharification with both alkaline or acidic pretreatments (Fan et al., 2020). Conversely, increasing *F5H* expression increases S lignin and saccharification in Arabidopsis after alkaline pretreatment (Ciesielski et al., 2014; Smith et al., 2022), or maleic acid (Sakamoto et al., 2020), or liquid hot water pretreatment (Li et al., 2010) and in poplar after pretreatments with alkali (Lapierre et al., 2021) or acid/alkali (Fan et al., 2020). In the rice *F5H1* CRISPR knock-out lines, saccharification efficiency was lowered compared to wild type only when using hot water pretreatment and unchanged with dilute acidic or alkaline pretreatments (Takeda et al., 2019b), whereas in switchgrass up- or down-regulation of *F5H* did not affect saccharification (Wu et al., 2019). In Brachypodium, *F5H* overexpression increased S lignin and saccharification without pretreatment (Sibout et al., 2017). In sugarcane, one F5H-suppressed line apparently had increased sugar release after mild acidic pretreatment but others did not (Bewg et al., 2016) so the relevance of this observation which conflicts with the rest of the literature is unclear. Our data showing that barley *HvF5H1-*RNAi plants have increased resistant phenylcoumaran units and reduced esterified *p*CA (which generally occurs as free-phenolic pendent groups on lignin (Ralph et al., 1994; Lu and Ralph, 1999)) suggests a more condensed or more *p*CA-decorated lignin would not necessarily have any beneficial effect on saccharification. Incidentally, in artificial model systems where other factors could be controlled, lignin S/G composition had little effect on wall degradability (Grabber et al., 1997; Grabber et al., 2009).

Downregulation of *HvF5H1 via* RNAi led to changes in the abundance of 173 features. The majority of metabolite features have unknown identities. As detailed in **Table S2**, 78 compounds were less abundant in the *HvF5H1*-RNAi plants. Predictably, S lignin-related compounds such as sinapyl alcohol and syringic acid 4-*O*-hexoside were among the known compounds shown to be decreased, which is consistent with the reduction of S lignin. The α‐oxidized β‐ether oligomer of sinapyl alcohol, Sox(8-O-4)S, was 150-fold less abundant in the *HvF5H1*-RNAi plants, followed by several small chains of G-S lignols such as G(8-O-4)S(8-8)S. Sox(8-O-4)S was previously shown also to be reduced 10-fold in abundance in barley *HvCOMT*-RNAi plants (Daly et al., 2019). On the other hand, the abundance of 95 features increased significantly in the *HvF5H1*-RNAi plants. Although the identity of the majority of these features is unknown, we notably found that di- or tri-lignols composed of only G units were increased 10 to 73-fold in the *HvF5H1*-RNAi lines, consistent with the increased abundance of G units in lignin and reduced proportion of S units. This is very different from *HvCOMT*-RNAi lines in which the greatest increased abundance detected involved 5‐hydroxyconiferyl alcohol, the product of F5H activity and substrate for subsequent COMT activity, and caffeoyl alcohol, another supposed substrate for COMT (Daly et al., 2019).

Although the G-unit-enriched lignin of *F5H*-suppressed plants may not be improved in digestibility, it may be useful for other purposes; for example the more highly-branched and less oxygenated lignin has a higher fuel-value and improved combustion properties. However, if we are to improve the lignocellulosic biomass of food crops it is crucial that changes in cell wall characteristics do not negatively affect agronomic traits. In this study, downregulation of *HvF5H1* did not lead to changes in grain characteristics or other agronomic traits and the mechanical characteristics of straw in the *HvF5H1*-RNAi lines were similar to the controls. Some previous studies have explored the relationship between lignin structure and biomechanical properties but firm conclusions have proved elusive. In poplar supressed in the lignin biosynthesis gene CAD, lignin content was associated with mechanical stiffness (Özparpucu et al., 2017), but the pronounced changes in lignin composition did not alter wood tensile properties (Özparpucu et al., 2018). However, in wheat, lignin content was not associated with straw breaking force but a lower proportion of S lignin appeared to increase it (Muhammad et al., 2020). In Arabidopsis *fah1* F5H mutants, stiffness was increased compared to wild type in apical and middle stem segments, but flexibility was unaltered between *fah1* and wild type plants (Ménard et al., 2022). Differences in stem architecture between dicots and grasses and indeed between individual species are likely to complicate any simplistic generalized interpretation of the effects of lignin characteristics on plant mechanical properties since even different morphotypes of tracheary element have different lignin chemistries and mechanical properties (Ménard et al., 2022). In our work, it is promising to see that radical changes to lignin composition and structure in HvF5H1 suppressed plants is not accompanied by any change to straw strength. Thus, F5H may represent a good target for manipulating lignin composition while maintaining crop health. Indeed, F5H suppression might improve some agronomic properties as illustrated recently in *Brassica napus* in which four genes for F5H were knocked out by CRISPR/Cas 9 and this apparently improved resistance to *Sclerotinia* stem rot (Cao et al., 2022).

Our work highlights once again that, despite striking similarities among grasses in terms of their cell wall composition, there may be important differences between species in their lignin biosynthetic pathways. For example, we previously showed that the key lignin biosynthetic gene caffeoyl shikimate esterase (CSE), originally identified in Arabidopsis, only seems to have *bone fide* orthologues in some grasses including rice and switchgrass, but is apparently absent in barley, Brachypodium, and maize (Vanholme et al., 2013; Ha et al., 2016). Here we show that the dominant route to S lignin biosynthesis in barley proceeds though the conventional lignin pathway and F5H, and that the proposed alternative ‘grass specific’ pathway that apparently exists in rice, is either absent or only a minor route in barley.

## 4 Materials and methods

### 4.1 Bioinformatics and phylogenetic analysis

Putative *HvF5H* genes were initially retrieved from a public database (Mayer et al., 2012) and reconfirmed with the latest released version (Mascher et al., 2017). Barley amino acid sequences of *Hv*F5H that showed 40% or more identity to the well-studied *At*FAH1 (Meyer et al., 1996) were selected. Next, the best aligning protein sequences were blasted back into the barley database to pull out putative genes with lower identity to *At*FAH1. Subsequently, all the selected protein sequences were aligned with those of functionally characterised *F5H* genes in rice (Takeda et al., 2017), switchgrass (Wu et al., 2019), sugarcane (Bewg et al., 2016), spike moss (Weng et al., 2008), Brachypodium (Sibout et al., 2017), maize (Andersen et al., 2008) and foxtail millet (Muthamilarasan et al., 2015). In the phylogenetic analysis we also used unpublished F5H sequences that stemmed from bioinformatics screenings. These genes were identified or revisited by blasting against the latest relevant genome database and selecting those containing all five signature motifs of F5Hs as described before (Chapple, 1998).

Phylogenetic analysis and construction of the tree were carried out using MEGA X software (Kumar et al., 2018). Amino acid sequences were aligned using ClustalW and a Neighbor-Joining tree constructed tested by 100 bootstrap replications. Evolutionary distances were estimated using the Poisson model based on the number of amino acid substitutions per site.

Barley genes transcriptomic profiling data from different plant tissues were extracted from the publicly available database (Mayer et al., 2012; Milne et al., 2021).

### 4.2 Plant materials, growth conditions, and designation of internodes

Barley cultivar Golden Promise was used to generate transgenic plants. All plants were grown in the greenhouse with light and temperature controls. High-pressure sodium lamps provided supplementary lights when necessary. Plants were grown in seven-inch pots filled with standard compost mix (150 L Levingtons M2 compost, 75 L perlite, 500 g Osmocote Plus Mini, 125 g Celcote Certis and 75 g Horti-Wet). Whole above-ground biomass of individual plants was harvested after senescence at the Z9.2 growth stage based on the Zadoks scale (Zadoks et al., 1974). Subsequently, biomass was air-dried at 35°C overnight. Following removing the leaves, second internodes from the main tillers were used to conduct cell wall characterization assays or saccharification. Second internodes were identified as described previously (Tottman, 1987).

### 4.3 Generation of barley *HvF5H1* RNAi transgenics

A cDNA of *HvF5H1* (*HORVU1Hr1G047220*) was used to amplify a 669 bp fragment using primers containing Gateway AttB sites. Subsequently, the amplicon was cloned into the entry vector using BP clonase II (Invitrogen) and then the fragments were transferred from the entry clone to the RNAi vector, pBract207 (John Innes Centre) using LR Clonase (Invitrogen). The RNAi cassette was transferred into *Agrobacterium tumefaciens* AGL1 containing the helper pSOUP plasmid. Barley transgenic lines were produced by the FunGen Group at The James Hutton Institute through the transformation of embryos of the Golden Promise cultivar (Harwood et al., 2009). The T0 transgenic lines were grown to produce seeds and perform preliminary screenings. The T1 generation was screened using a hygromycin root assay to establish plants’ zygosity and the southern blot assay to determine numbers of T‐DNA loci. Briefly, twenty seeds were germinated on agar plates with 0.5% phytogel and 100 µg/mL of hygromycin as described by Jacobsen et al. (2006). In subsequent generations, hygromycin assays and PCR was used on germinating progeny from parental plants to identify parents homozygous for the RNAi transgene and those that had lost it through segregation (azygotes).

### 4.4 Expression analysis in RNAi lines

RNA extraction was performed using TRI-Reagent (Sigma-Aldrich) as described by the manufacturer. The resulting RNA was further cleaned up using the Qiagen RNeasy Mini Kit RNA Cleanup Protocol (Cat No 74536). 500 ng of RNA was used to synthesise cDNA using qScript (Perfecta). The resulting products contain a supermix that has both OligoDT and random primers for better amplification. This was then amplified using the protocol outlined by the manufacturer. The expression analysis was conducted using the Applied Biosystems StepOne machine and related software. SnRK1 (SNF1-related kinase 1) was selected as the internal control gene and we used primers designed against the 3 different orthologs of the *HvF5H1* gene. Primers and probes were designed (allocated) through the Roche Universal Probe Library version.

### 4.5 Mäule staining

Freehand stem cross sections were obtained from the second internodes using a No. 11 Scalpel blade. Sections were fixed in 4% glutaraldehyde for 1 h at room temperature, rinsed with water, and immersed in 0.5% (w/v) potassium permanganate solution for 10 min at room temperature. Then they were rinsed with water several times and incubated in 10% hydrochloric acid for 5 min. Following rinsing twice with water, the sample was dipped in concentrated ammonium hydroxide under a fume hood for 2 min and mounted on the slide. A Nikon light microscope was employed to study stem sections. Images were taken using a microscope mounted Lecia DC500 camera.

### 4.6 Klason lignin and lignin composition

Lignin contents were quantified following modification of a previously described method (Dence, 1992). In summary, whole tillers (excluding sheaths and leaves) of three biological repeats for each line were powdered to 0.5 mm particle sizes using a Retsch ZM-200 centrifugal rotor mill and dried at 35°C overnight. Followed by an extraction process using exhaustive hot water and hot ethanol *via* Soxhlet. Samples of 300 ± 5 mg were then hydrolysed in 5 mL of 72% H_2_SO_4_ for 2 h. Distilled water (12.5 mL) was added and the samples were kept at room temperature for 1 h before being transferred into 250 mL Schott bottles by washing with 150 mL dH_2_O. The samples were incubated at 121°C for 1 h and filtered through a crucible to collect both the solid (acid-insoluble fraction) and the liquid filtrate (acid-soluble fraction). The lignin was collected by vacuum filtration on Whatman GF/C 55 mm filter paper and subsequently washed and dried at 110°C overnight. The lignin content was calculated as a percentage of the dry weight of the starting material. Lignin values were adjusted for ash content by burning the extracted lignin at 545°C in a furnace.

Lignin structure was evaluated by the simplified thioacidolysis procedure as described previously (Lapierre et al., 2021). Ester-linked *p*-coumaric and ferulic units were measured after mild alkaline hydrolysis as described previously (Sibout et al., 2016).

### 4.7 Cell wall characterization by two-dimensional solution-state NMR

Cell walls were characterised without fractionation using two-dimensional (2D) solution-state NMR (Kim and Ralph, 2010; Mansfield et al., 2012). Straw (2-mm pieces) was pre-ground using a Mixer Mill MM400 (Retsch; 30/s vibrational frequency for 90–120 s). Samples were extracted three times with water, three times with 80% ethanol and once with acetone, then allowed to dry. The pre-ground extracted samples were ball-milled using a Fritsch Planetary micro mill Pulverisette 7 vibrating at 600 rpm with zirconium dioxide (ZrO_2_) vessels containing ZrO_2_ ball-bearings (10 mm × 10) with 5-min milling and 5-min cooling per milling cycle (cycle number depended on the amount of sample). The ball-milled samples were subjected to digestion (72 h × 2) to obtain ‘enzyme lignin’ (EL) by Cellulysin^®^ cellulase from *Trichoderma viridae* (Calbiochem), at 35°C in acetate buffer (pH 5.0). The ELs were dissolved into DMSO-d_6_/pyridine-d_5_ (4:1 v/v) and subjected to NMR on a Bruker Biospin AVANCE-III 700 MHz spectrometer equipped with a 5-mm QCI ^1^H/^31^P/^13^C/^15^N cryoprobe with inverse geometry (proton coil closest to the sample). 2D-^1^H–^13^C HSQC spectra were acquired using Bruker’s pulse program (hsqcetgpsip2.2). Bruker’s Topspin 3.2 (Mac) software was used to process spectra. The central DMSO peak was used as internal references (δ_C_: 39.51, δ_H_: 2.49 ppm).

### 4.8 Transcript and metabolite profiling

Five plants from each line were grown for 61 days in a Randomised Block Design (RBD) in a greenhouse with supplementary lighting. The first three internodes of each plant were collected and ground in liquid nitrogen, subsequently 100 mg of the ground stem from each replication was used for transcriptome or metabolite analysis as described before (Daly et al., 2019).

### 4.9 Straw mechanical properties

Straw was sampled from the RNAi and control lines at harvest time, Zadoks stage 97 (Zadoks et al., 1974), and mechanical properties were analysed by quantifying the Flexural Strength Stress at Yield. Second and fourth internodes from the main tiller were fixed into in an Instron Universal Testing System (https://www.instron.com/) and the force needed to break the straw was measured.

### 4.10 Saccharification analyses

A robotic platform (Labman Automation Ltd, UK) was employed for alkaline pre-treatment and saccharification as described before (Oakey et al., 2013). A modified version was used for hot water pre-treatment and subsequent saccharification. Briefly, 7.0 ± 0.5 mg extracted pulverised sample was pre-treated with hot water in the autoclave at 130°C for 30 min. The solid was washed 3× with deionised water. The samples were then incubated while shaking at 50°C for 96 h in the presence of an enzyme cocktail (4:1 ratio of Celluclast and Novozyme 188). The GOPOD assay kit (K-GLUC) (Megazyme, Ireland) was used to quantify the glucose released during saccharification.

### 4.11 Statistical analysis

Data were collected as described for each trait. Where required, a square root transformation of response was used to ensure model assumptions of normality and homogeneity of variance in residuals were met (Miller, 1997). T-test was employed when comparing two groups. To compare significance levels for three or more groups, ANOVA was employed. Where ANOVA showed significance at the 5% level, *post-hoc* Tukey’s test was used for comparisons. The Minitab Statistical Software version 18.1 or R (R Core Team, 2021) was used for statistical analysis. Model-adjusted means were used for the metabolomics/transcriptomic experiment. Metabolites/probes for which the combined mean was at least threefold and significantly (P<0.01) different from combined controls in both *HvF5H1*-RNAi lines were considered to be significantly different.

## Data availability statement

The datasets presented in this study can be found in online repositories. The names of the repository/repositories and accession number(s) can be found in the article/supplementary material. Additionally, the reference transcript dataset (RTD) is available in the Zenodo repository https://doi.org/10.5281/zenodo.3360434.

## Author contributions

RS performed phylogenetic analysis, designed genetic constructs and, along with MH, characterized the transgenic plants. JS produced the transgenic plants. CL performed thioacidolysis and mild alkaline hydrolysis. YT and JR performed 2D NMR. CC and HO designed the metabolomic and transcriptomic experiments. GG, RV and WB performed the metabolomics analysis. CH conceived and coordinated the experiments. RS and CH wrote the manuscript. All authors contributed to the article and approved the submitted version.
