# Supplementary material for: Downregulation of barley ferulate 5-hydroxylase dramatically alters straw lignin structure without impact on mechanical properties
Source: Front Plant Sci. 2023 Jan 16;13:1125003. doi: 10.3389/fpls.2022.1125003 (PMC9886061; doi:10.3389/fpls.2022.1125003)
Supplement: Supplementary file 1 [file Presentation_1.pptx]

## Slide 1
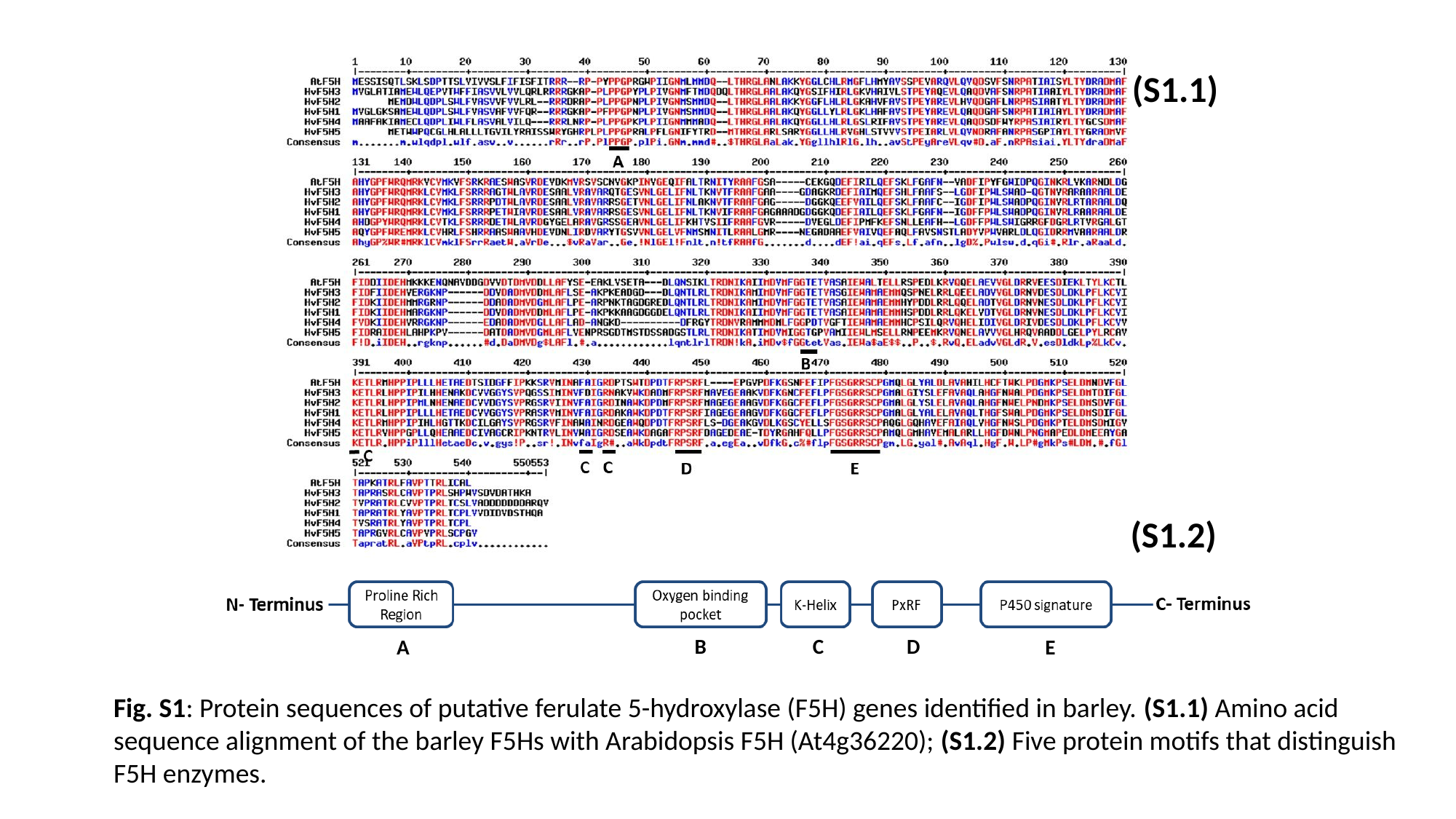

(S1.1)
(S1.2)
B
C
D
A
E
Fig. S1: Protein sequences of putative ferulate 5-hydroxylase (F5H) genes identified in barley. (S1.1) Amino acid sequence alignment of the barley F5Hs with Arabidopsis F5H (At4g36220); (S1.2) Five protein motifs that distinguish F5H enzymes.

## Slide 2
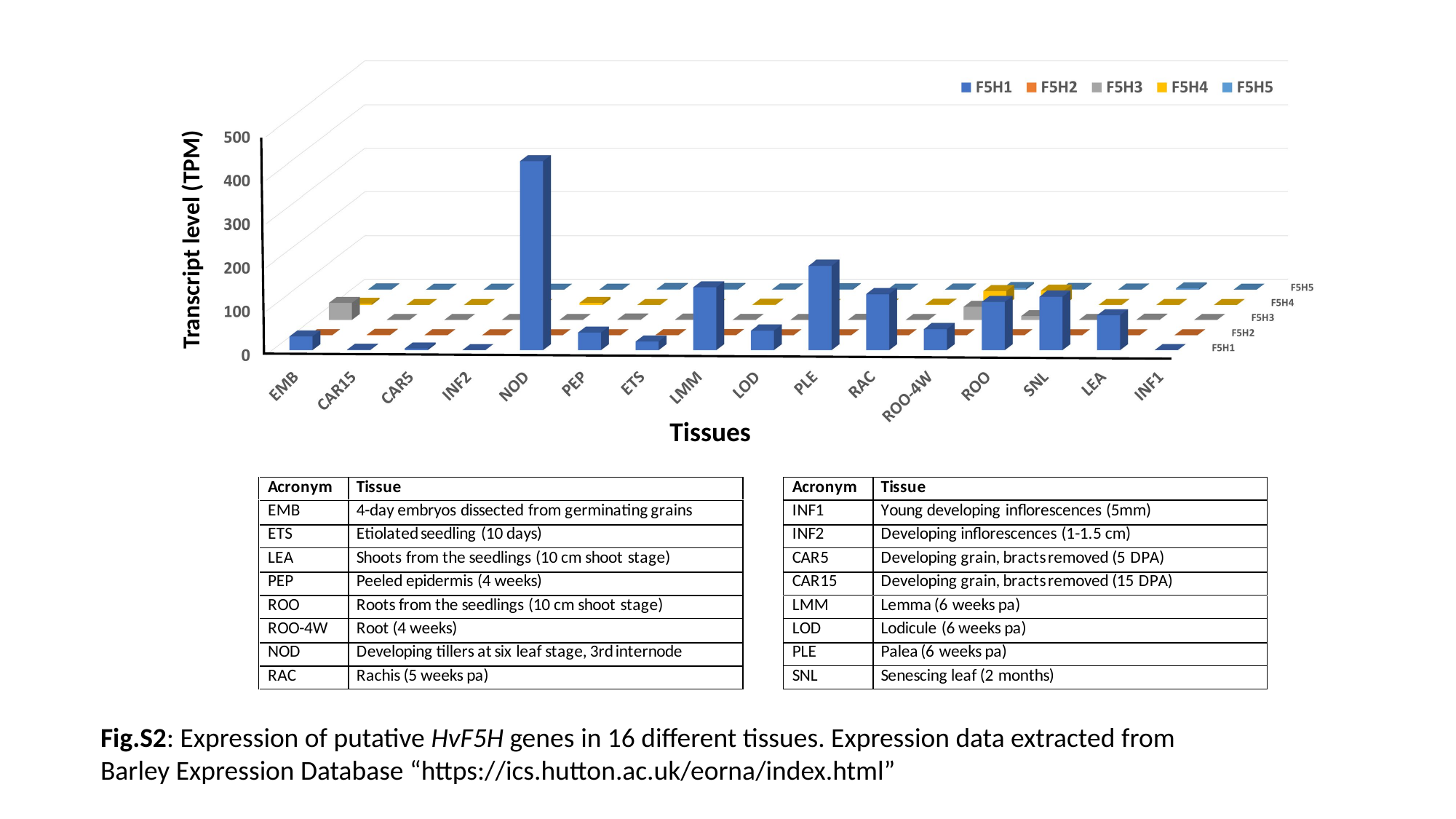

Transcript level (TPM)
Tissues
Fig.S2: Expression of putative HvF5H genes in 16 different tissues. Expression data extracted from Barley Expression Database “https://ics.hutton.ac.uk/eorna/index.html”

## Slide 3
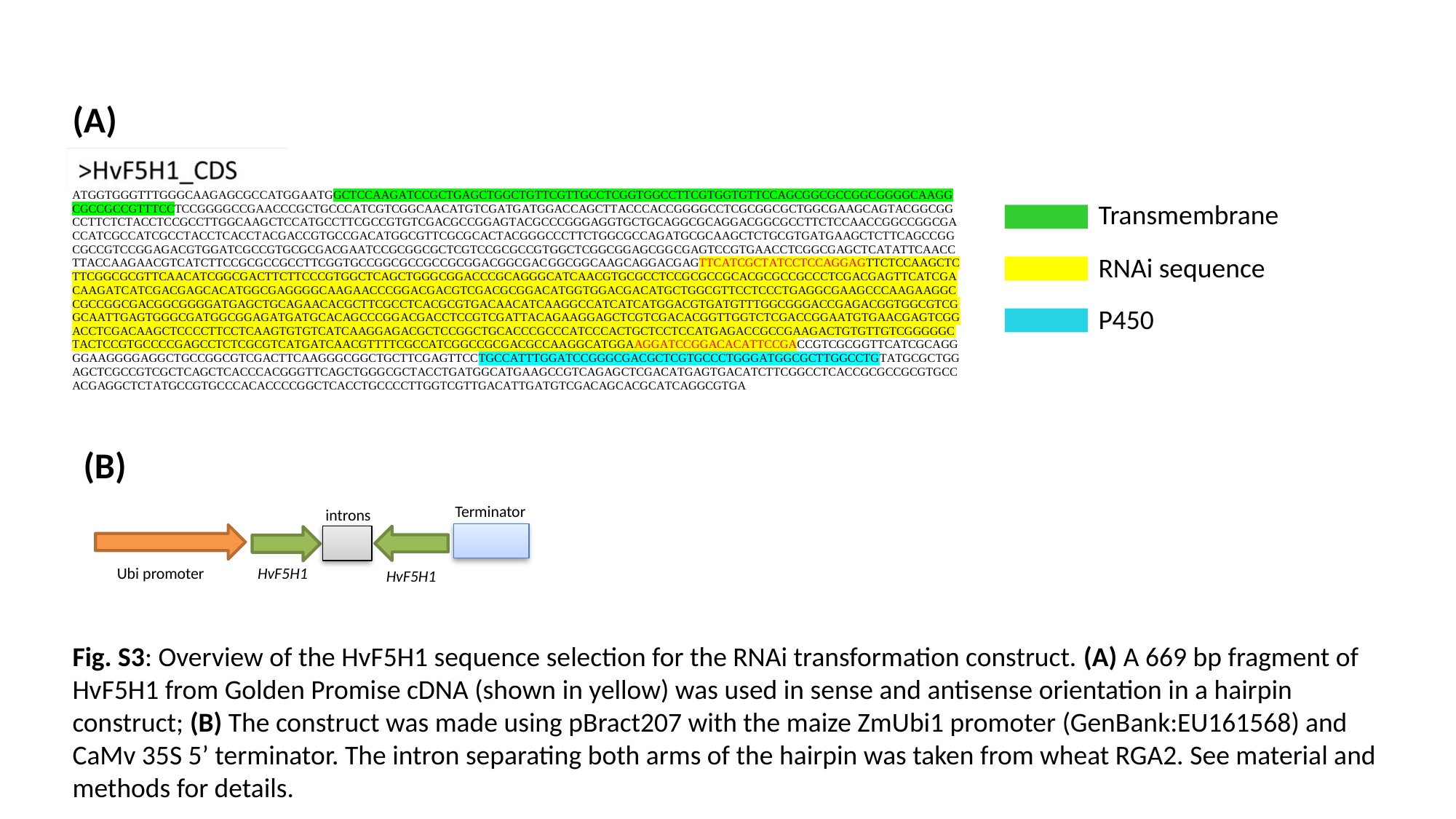

(A)
Transmembrane
RNAi sequence
P450
(B)
Terminator
 introns
Ubi promoter
HvF5H1
HvF5H1
Fig. S3: Overview of the HvF5H1 sequence selection for the RNAi transformation construct. (A) A 669 bp fragment of HvF5H1 from Golden Promise cDNA (shown in yellow) was used in sense and antisense orientation in a hairpin construct; (B) The construct was made using pBract207 with the maize ZmUbi1 promoter (GenBank:EU161568) and CaMv 35S 5’ terminator. The intron separating both arms of the hairpin was taken from wheat RGA2. See material and methods for details.

## Slide 4
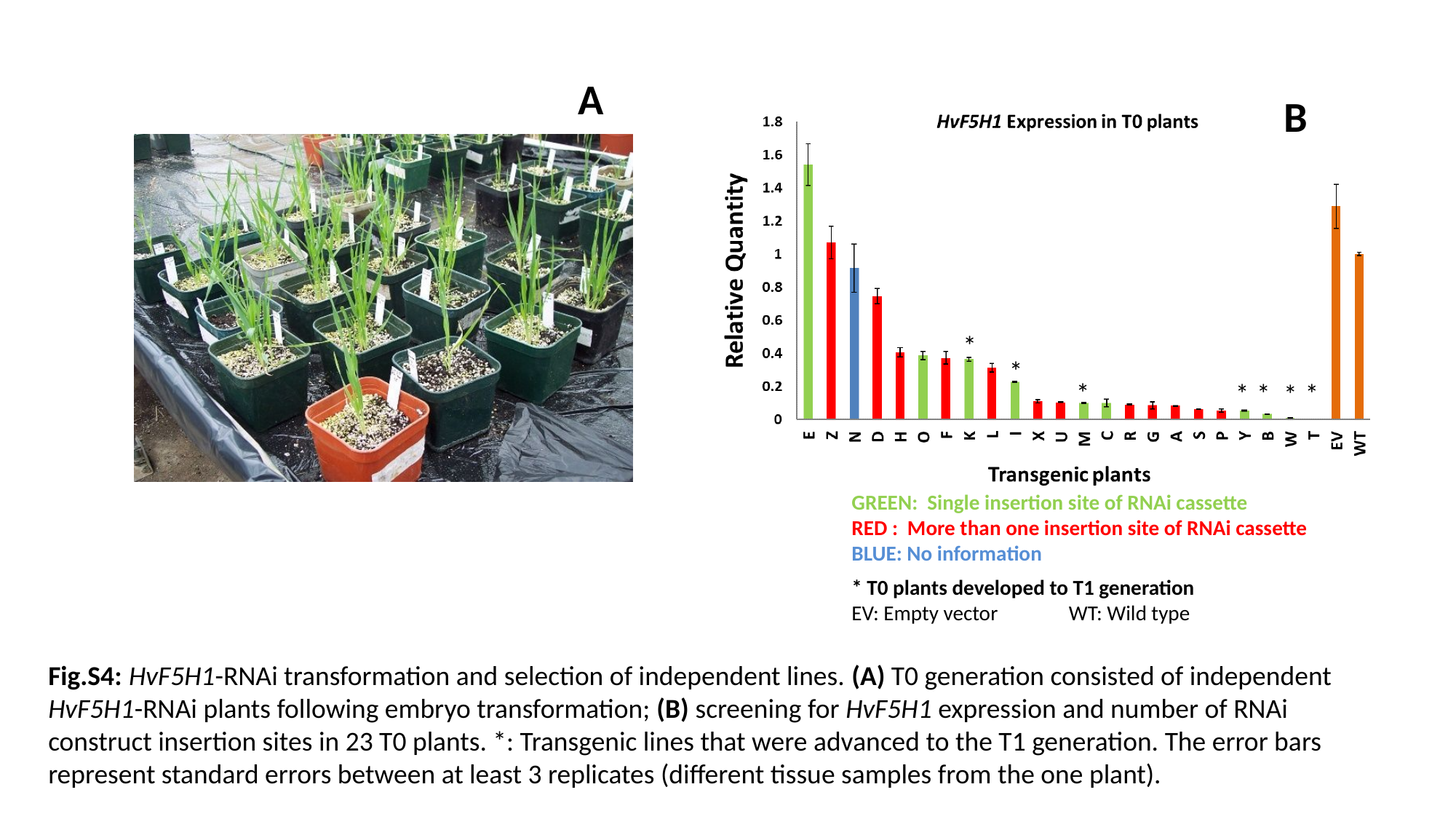

A
B
*
*
*
*
*
*
*
GREEN: Single insertion site of RNAi cassette
RED : More than one insertion site of RNAi cassette
BLUE: No information
* T0 plants developed to T1 generation
EV: Empty vector WT: Wild type
Fig.S4: HvF5H1-RNAi transformation and selection of independent lines. (A) T0 generation consisted of independent HvF5H1-RNAi plants following embryo transformation; (B) screening for HvF5H1 expression and number of RNAi construct insertion sites in 23 T0 plants. *: Transgenic lines that were advanced to the T1 generation. The error bars represent standard errors between at least 3 replicates (different tissue samples from the one plant).

## Slide 5
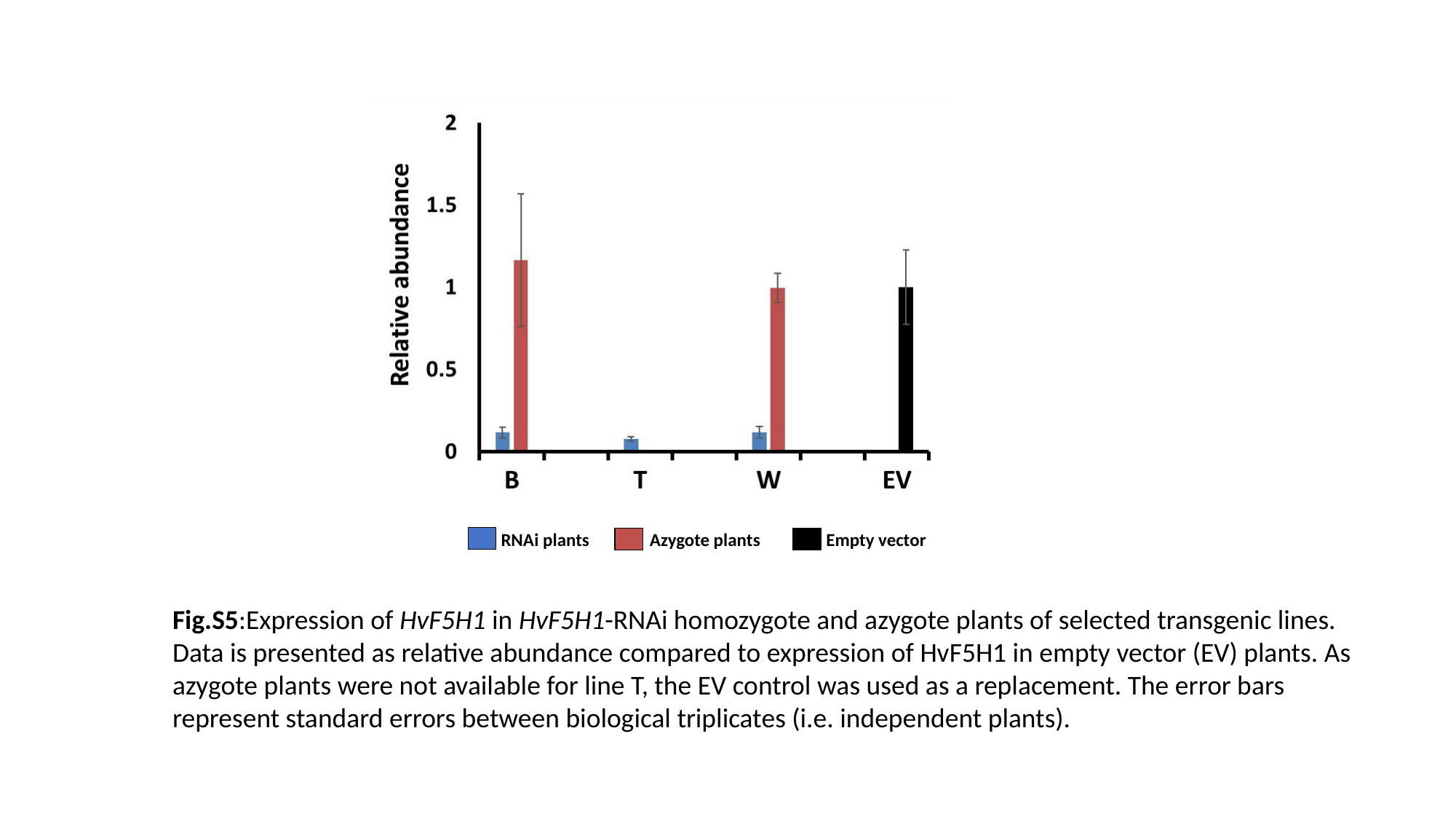

RNAi plants
Azygote plants
Empty vector
Fig.S5:Expression of HvF5H1 in HvF5H1-RNAi homozygote and azygote plants of selected transgenic lines. Data is presented as relative abundance compared to expression of HvF5H1 in empty vector (EV) plants. As azygote plants were not available for line T, the EV control was used as a replacement. The error bars represent standard errors between biological triplicates (i.e. independent plants).

## Slide 6
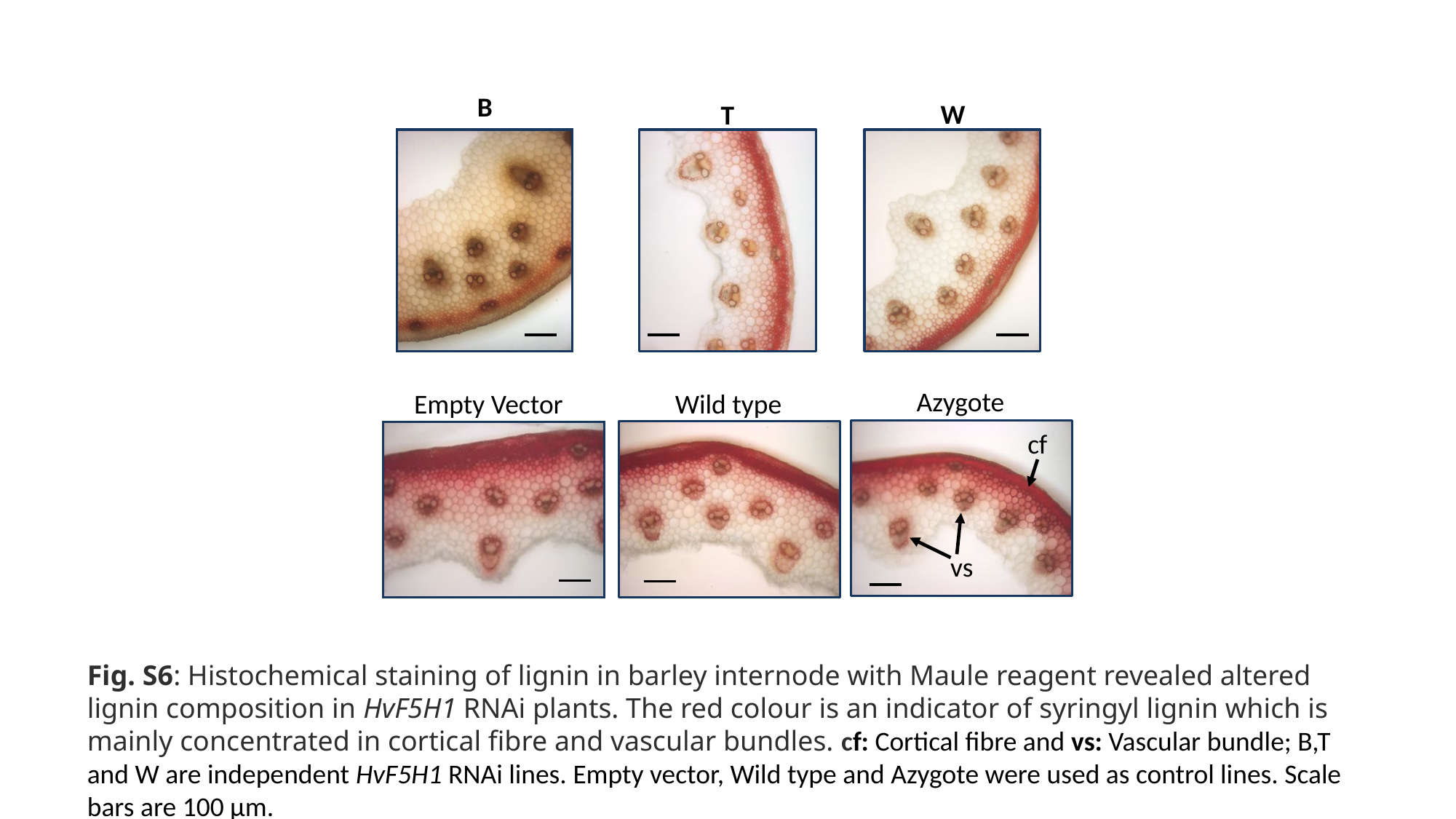

B
W
T
Azygote
Empty Vector
Wild type
cf
vs
Fig. S6: Histochemical staining of lignin in barley internode with Maule reagent revealed altered lignin composition in HvF5H1 RNAi plants. The red colour is an indicator of syringyl lignin which is mainly concentrated in cortical fibre and vascular bundles. cf: Cortical fibre and vs: Vascular bundle; B,T and W are independent HvF5H1 RNAi lines. Empty vector, Wild type and Azygote were used as control lines. Scale bars are 100 μm.

## Slide 7
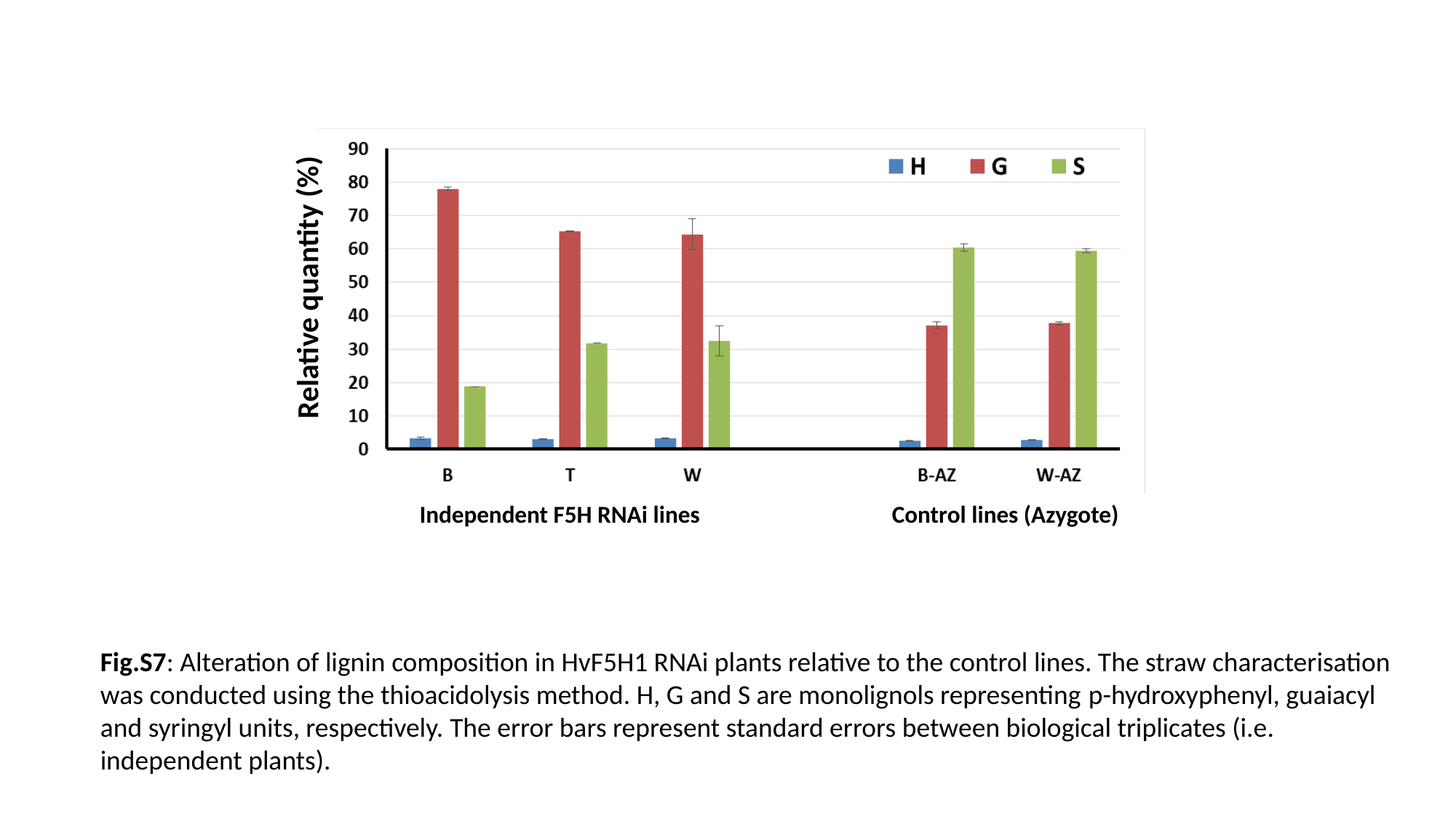

Relative quantity (%)
Independent F5H RNAi lines
Control lines (Azygote)
Fig.S7: Alteration of lignin composition in HvF5H1 RNAi plants relative to the control lines. The straw characterisation was conducted using the thioacidolysis method. H, G and S are monolignols representing p-hydroxyphenyl, guaiacyl and syringyl units, respectively. The error bars represent standard errors between biological triplicates (i.e. independent plants).
