# Supplementary material for: Downregulation of barley ferulate 5-hydroxylase dramatically alters straw lignin structure without impact on mechanical properties
Source: Front Plant Sci. 2023 Jan 16;13:1125003. doi: 10.3389/fpls.2022.1125003 (PMC9886061; doi:10.3389/fpls.2022.1125003)
Supplement: Supplementary file 3 [file Table_2.docx]

**Table S4.** Agronomic and seed characteristics of HvF5H1-RNAi and controls (corresponding azygote lines, empty vector and wild type)

| **Genotypes** | **Shoot height (cm)** | **Tiller No.** | **Straw biomass (g)** | **Spike No.** | **TGW (g)** | **Seed width (mm)** | **Seed length (mm)** | **Seed area (mm^2^)** |
| --- | --- | --- | --- | --- | --- | --- | --- | --- |
| **B_F5H1 RNAi** | 48.2 ± 0.86 ^a^ | 15.8 ± 3.02^ab^ | 10.06 ± 1.5 ^a^ | 14.8 ± 3.28 ^ab^ | 24.4 ± 1.30 ^a^ | 2.8 ± 0.06 ^a^ | 8.62 ± 0.05 ^a^ | 19.27 ± 0.28 ^a^ |
| **B_Azygote** | 50 ±1.87 ^a^ | 12.4 ± 1.03 ^b^ | 9.55 ± 1.08 ^a^ | 11.2 ± 0.66 ^ab^ | 24.2 ± 1.28 ^a^ | 2.8 ± 0.06 ^a^ | 8.45 ± 0.1 ^a^ | 18.65 ± 0.31 ^a^ |
|  |  |  |  |  |  |  |  |  |
| **T_F5H1 RNAi** | 51.2 ± 4.05 ^a^ | 11.0 ± 1.76^b^ | 8.83 ± 1.35 ^a^ | 10.2 ± 1.96 ^b^ | 23.9 ± 1.62^a^ | 2.8 ± 0.06 ^a^ | 8.53 ± 0.09 ^a^ | 18.57 ± 0.29 ^a^ |
|  |  |  |  |  |  |  |  |  |
| **W_F5H1 RNAi** | 52.4 ± 1.63 ^a^ | 20.0 ± 1.12 ^ab^ | 12.67 ± 1.74 ^a^ | 15.4 ± 1.81 ^ab^ | 23.7 ± 1.77 ^a^ | 2.8 ± 0.06 ^a^ | 8.52 ± 0.09 ^a^ | 18.43 ± 0.36 ^a^ |
| **W_ Azygote** | 44.2 ± 3.53 ^a^ | 23.0 ± 3.9 ^a^ | 11.97 ± 2.19 ^a^ | 21.2 ± 3.61 ^a^ | 24.3 ± 1.67 ^a^ | 2.8 ± 0.08 ^a^ | 8.42 ± 0.05 ^a^ | 18.35 ± 0.31 ^b^ |
|  |  |  |  |  |  |  |  |  |
| **EV** | 48.6 ± 3.42 ^a^ | 16.2 ± 3.56 ^ab^ | 10.13 ± 1.49 ^a^ | 15.2 ± 3.48 ^ab^ | 28.4 ± 5.07 ^a^ | 2.9 ± 0.17 ^a^ | 8.66 ± 0.12 ^a^ | 19.58 ± 1.21 ^a^ |
| **WT** | 44.4 ± 2.42 ^a^ | 14.4 ± 2.33 ^ab^ | 9.79 ± 0.85 ^a^ | 14 ± 2.35 ^ab^ | 21.1 ± 0.51 ^a^ | 2.6 ± 0.03 ^a^ | 8.52 ± 0.03 ^a^ | 17.67 ± 0.14 ^a^ |

Values are means ± SEM of five replications, data were analysed in a two-way ANOVA and the TUKEY test performed for mean analysis (P < 0.05), means that do not share a letter are significantly different. EV: Empty vector; WT: wild type.

**Table S5.** Stem mechanical properties of HvF5H1-RNAi and controls (corresponding azygote lines, empty vector and wild type)

| **Genotypes** | **2^nd^ internode diameter (mm)** | **4^th^ internode diameter (mm)** | **Flexural strength at Yield (2^nd^ internode), MPa** | **Flexural strength at Yield (4^th^ internode), MPa** |
| --- | --- | --- | --- | --- |
| **B_F5H1 RNAi** | 3.12 ± 0.23 ^ab^ | 3.20 ± 0.15 ^ab^ | 19.18 ± 4.2 ^a^ | 11.54 ± 2.8 ^a^ |
| **B_Azygote** | 2.70 ± 0.19 ^ab^ | 2.48 ± 0.08 ^b^ | 18.91 ± 5.1 ^a^ | 15.85 ± 0.84 ^a^ |
|  |  |  |  |  |
| **T_F5H1 RNAi** | 3.27 ± 0.09 ^ab^ | 3.25 ± 0.22 ^ab^ | 17.33 ± 2.22 ^a^ | 15.65 ± 4.33 ^a^ |
|  |  |  |  |  |
| **W_F5H1 RNAi** | 3.32 ± 0.20 ^a^ | 3.21 ± 0.23 ^ab^ | 17.13 ± 1.96 ^a^ | 13.83 ± 0.87 ^a^ |
| **W_ Azygote** | 3.13 ± 0.10 ^ab^ | 3.24 ± 0.13 ^ab^ | 15.92± 4.26 ^a^ | 10.91 ± 3.62 ^a^ |
|  |  |  |  |  |
| **EV** | 2.95 ± 0.11 ^ab^ | 3.18 ± 0.04 ^ab^ | 20.05 ± 2.49 ^a^ | 12.06 ± 0.12 ^a^ |
| **WT** | 2.91 ± 0.18 ^ab^ | 2.76 ± 0.10 ^ab^ | 16.26 ± 1.48 ^a^ | 14.04 ± 0.72 ^a^ |

Values are means ± SEM of three replications, data were analysed in a two-way ANOVA and the TUKEY test performed for mean analysis (P < 0.05), means that do not share a letter are significantly different. EV: Empty vector; WT: wild type.
